# Anesthetic Diffusion Through Lipid Membranes Depends on the Protonation Rate

**DOI:** 10.1038/srep07534

**Published:** 2014-12-18

**Authors:** Rosendo Pérez-Isidoro, F. J. Sierra-Valdez, J. C. Ruiz-Suárez

**Affiliations:** 1 CINVESTAV-Monterrey, PIIT, Nuevo León, 66600, México

## Abstract

Hundreds of substances possess anesthetic action. However, despite decades of research and tests, a golden rule is required to reconcile the diverse hypothesis behind anesthesia. What makes an anesthetic to be local or general in the first place? The specific targets on proteins, the solubility in lipids, the diffusivity, potency, action time? Here we show that there could be a new player equally or even more important to disentangle the riddle: the protonation rate. Indeed, such rate modulates the diffusion speed of anesthetics into lipid membranes; low protonation rates enhance the diffusion for local anesthetics while high ones reduce it. We show also that there is a pH and membrane phase dependence on the local anesthetic diffusion across multiple lipid bilayers. Based on our findings we incorporate a new clue that may advance our understanding of the anesthetic phenomenon.

Alkanes^1^,^2^, alcohols^3^,^4^,^5^, benzodiacepines^6^,^7^, barbiturates^7^,^8^, esters and amides^9^,^10^,^11^, phenols^7^,^12^, ethers^13^,^14^,^15^ and even inert gases^15^,^16^ are anesthetics^17^. Nonetheless, despite the vast diversity of studies, a theory able to explain all aspects behind anesthesia is still unfinished. Two theories struggle to explain it: one professes that the drug acts on lipids, the other on proteins^18^,^19^,^20^,^21^,^22^,^23^. Both achieve high notes of success although leave untenable important issues^24^. Anesthetic molecules have been used for local or general anesthesia based on their pharmacokinetic and toxicological effects, but only very few have been prevailed in clinical practice^25^,^26^,^27^. One of the most relevant pharmacokinetic concerns is how the drugs diffuse in tissues, which for either general or local anesthetics depend on lipid-solubility, i.e., its partition coefficient. Moreover, for local anesthetics the diffusivity is pH dependent^28^,^29^. General^30^,^31^,^32^ and local anesthetics^33^,^34^, even with its pH dependence^35^, have been demonstrated to depress the melting point transition of lipid membranes, which has been also correlated with the partition coefficient. The latter drugs are weak bases with three concomitant components: (a) a lipophilic aromatic ring, (b) an intermediate ester or amide chain, and (c) a terminal amine. The first two determine the lipophilic aspects of the molecule, in which greater lipid solubility enhances diffusion through nerve sheaths toward the neural membrane. This property correlates with drug potency. In aqueous solution, the terminal amine acts as an “on-off switch”, where depending on pH, allows the local anesthetic to exist in a tertiary form (non-protonated); that is, lipid soluble or as a quaternary form (protonated) that is positively charged and therefore water soluble. It is assumed that the protonated form is related to the anesthetic-protein interaction^10^,^11^. Indeed, since non-protonated species present faster diffusion in tissues, alkalinisation of local anesthetic solutions reduces the pain of infiltration at the expenses of reducing the onset and duration of anesthesia^36^,^37^. Each one, protonated and non-protonated species, plays a crucial role in the succession of events leading to conduction blocking. All in all, there are not physical and chemical evidences supporting the accepted framework of local anesthetic diffusion through lipid membranes, i.e., how the change rate between protonated and non-protonated species modulate it. In this work, using differential scanning calorimetry (DSC), we investigated some critical diffusive aspects of local anesthetics through a temporal study of the phase transition changes of pure lipid membranes. This analysis was compared with general anesthetics; both intravenous and inhalational. Firstly, our results show that in clinical conditions the diffusion of local anesthetics is slower than general anesthetics. Additionally, our data give the first evidence of the membrane phase dependence of drug diffusion. A thorough analysis indicates a pH dependence of local anesthetic diffusion, revealing an interesting behavior precisely at pH range of clinical application. Focusing on this pH, an additional analysis suggests that the use (or not) of different carboxylic acids in solution, and the chemical nature, induces significant differences in the diffusive kinetics of local anesthetics across multiple bilayers. Our results demonstrate, for the first time, the importance of the protonation rate on the interlamellar diffusive kinetics of local anesthetics, which in comparison with general anesthetics, allows us to illustrate the inherent essence of local or general anesthetic function.

## Results

### A ‘double-phase transition’ discloses an intermediate state in the local anesthetic diffusion in MLV

**Figure 1:**
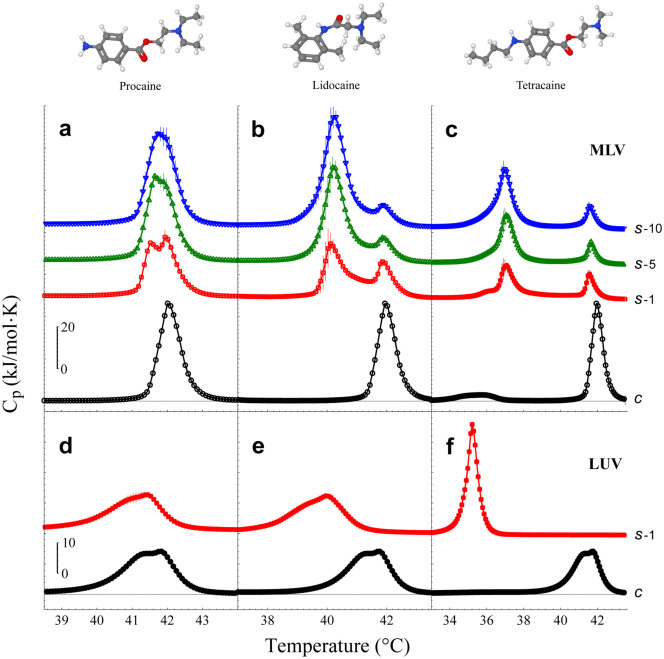
Calorimetric profiles of MLV and LUV systems under the influence of local anesthetics. Local anesthetics were added to the MLV and LUV suspension at their respective clinical concentration. After 10 min, a complete sequence of successive heating scans was performed. (a) Procaine (73 mM), (b) Lidocaine (69 mM) and (c) Tetracaine (25 mM). Control experiments (c, black circles) showed the main transition temperature at 42.0°C ± 0.01°C. Scans 1 (*s-1*, red squares), 5 (*s-5*, green up-triangles) and 10 (*s-10*, blue down-triangles) are taken from the scan series. The time between scans is about 36 min. The same process is shown for (d) Procaine, (e) Lidocaine and (f) Tetracaine in a LUV suspension. Since all the subsequent LUV scans do not show differences, only scan 1 (*s-1*, red squares) is presented. Intrinsic differences between MLV and LUV are commonly observed in the control calorimetric profiles, however, ‘clinical conditions’ were responsible for this peculiar deformation in the main transition of LUV. The solution was adjusted to pH ~5 (HCl/NaOH). Error bars show standard deviation.

Diffusion of anesthetics is an outstanding property that determines their respective clinical use^25^. DSC has been used to model the drug permeation kinetics into and through lipid membranes^38^,^39^. However, our results suggest a new method to pursue the diffusion process, which is based on calorimetric enthalpy measurements. To assess the physicochemical aspects supporting anesthetic diffusivity, we investigate the diffusion process of three local anesthetics, procaine (PCN), lidocaine (LCN) and tetracaine (TCC), across both multilamellar (MLV) and unilamellar (LUV) vesicles composed of 1,2-dipalmitoyl-*sn*-glycero-3-phosphocholine (DPPC) (Fig. 1). Experiments were performed under ‘clinical conditions’, comprising: ultrapure water pH 4–6, 100 mM NaCl and 0.97 µM Phenylephrine (Phe)^37^. As expected, both MLV and LUV systems are equally affected by the respective local anesthetic clearly observed through the main-transition temperature depression (ΔT_m_).

However, experiments with local anesthetics in MLV show a ‘double-phase transition’ (Fig. 1a,b,c), while in LUV a ‘single-phase transition’ is observed (Fig. 1d,e,f). The ‘double-phase transition’ can be explained by considering two different states. The first one corresponds to outer membranes affected by the anesthetics (left peak), while the second corresponds to inner membranes that have not been perturbed yet (right peak). Regardless the number of lipid bilayers contained in the multilamellar liposomes, two different states are always observed. It is important to remark that the melting transition shift induced by TCC is concentration dependent, which should imply intermediate transitions due to the gradual penetration of TCC through inner bilayers. However, intriguingly, only two peaks are observed. We believe that the external membranes of the MLV system immediately reach a maximum TCC concentration (saturated state), which can not be surpassed for a given TCC concentration in the aqueous medium. Thus, such saturated TCC concentration on membranes depends strictly on the TCC concentration of the aqueous medium. This results in a small peak that is shifted with respect to the original (control) peak, i.e., left peak. Thereafter, the drug contained inside such outer membranes, diffuses inward, momentarily depleting its concentration but being compensated fast by the TCC of the aqueous medium (in other words, the outer membranes are in equilibrium). Due to the polydispersity of the MLV system, the subsequent inner bilayers do not contain the same TCC concentration at the time of the measurement and therefore these enthalpy contributions are not enough to generate a continuum. Once the inner bilayers gradually get the saturated state, their enthalpy contributes to the left peak, explaining thus how it increases through the DSC scans. This might be the reason for having only two narrow peaks and not a broad signal.

Experiments where the local anesthetic was added during the hydration process of lipids in the MLV preparation, do exhibit the same results as LUV preparation; in other words, only one peak is present (Supplementary Fig. S1), confirming that a ‘double-phase transition’ corresponds to an intermediate stage of the drug diffusion kinetics across lipid membranes. Additional control experiments demonstrated that Phe does not perturb the DPPC membranes at such clinical concentrations (data not shown in this work). However, we explored the effect of Phe only in the ‘clinical conditions’ experiments.

The corresponding clinically used concentrations are within the saturation regimen of ΔT_m_ (data not shown), so that their ΔT_m_ are comparable. Irrespective of ‘double’ or ‘single’ phase transition, the order of ΔT_m_ was TCC>LCN>PCN, which was consistent with the order of their hydrophobicities and the anesthetic potencies^28^,^34^,^41^,^42^.

These data therefore indicate that, in ‘clinical conditions’, the time scale for local anesthetics diffusion is relatively slow to perform a deeper analysis into their diffusion kinetics.

### A ‘single-phase transition’ in MLV suggests a faster general anesthetic diffusion

**Figure 2:**
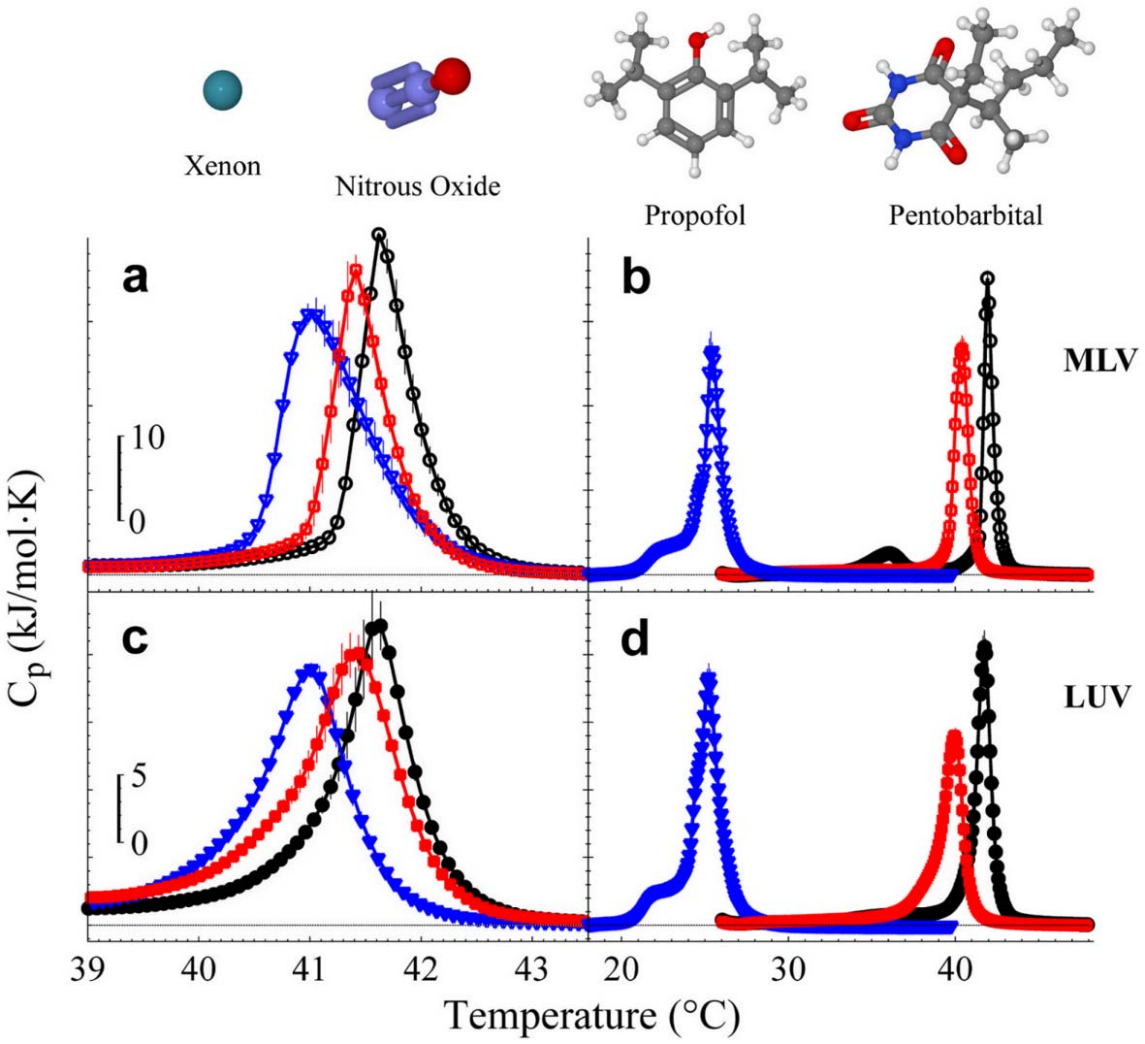
Calorimetric profiles of MLV and LUV systems under the influence of general anesthetics. Inhalational anesthetics were introduced to MLV and LUV under a high-pressure system (HPS) at 40 atm, 70°C, during 2 hrs. After this pressurizing process, a heating scan is taken by the DSC. Inhalational anesthetics, Xe (red squares) and N_2_O (blue triangles) in (a) MLV and (c) LUV. Intravenous anesthetics were added to the MLV and LUV suspension to their respective concentrations, 56 mM (PPF) and 25 mM (PB). After 10 min the heating scan is started. Intravenous anesthetics, PB (red squares) and PPF (blue triangles) in (b) MLV and (d) LUV. Control experiments (black circles) showed the main transition temperature at 41.8°C ± 0.01°C. The solution was adjusted to pH ~5 (HCl/NaOH). Error bars represent standard deviation.

To compare the diffusivity between local and general anesthetics across DPPC membranes, DSC experiments with inhalational (nitrous oxide, N_2_O and xenon, Xe) and intravenous (pentobarbital, PB and propofol, PPF) were carried out. Experiments were performed in ultrapure water with the exception of pentobarbital, which was obtained by injectable solution. The results with inhalational (Fig. 2a,c) and intravenous (Fig. 2b,d) anesthetics, illustrate a ‘single-phase transition’ in both MLV and LUV.

For intravenous anesthetics, the ΔT_m_ was greater for PPF than PB. On the other hand, the ΔT_m_ was greater for Xe than N_2_O, both applied under the same conditions (40 atm). The innate hydrophobicity of inhalational anesthetics prohibits their solubility in the aqueous media of the liposome suspension, by which high pressures were required to increase water solubility through Henry's law. The high pressures used are necessary to reach the saturation regimen of ΔT_m_ induced by Xe^32^ and N_2_O (data not shown). The experiments with inhalational anesthetics, supplied under a high-pressure system (HPS), are not performed as clinically done, since high pressures are needed to increase water solubility of both gases. Independently, our results with both intravenous and inhalational in their respective conditions are also consistent with the order of their hydrophobicities and the anesthetic potencies^43^,^44^. Accordingly, a ‘single-phase transition’ observed in MLV allows us to infer that, general anesthetics present faster diffusivity. Molecular arguments behind the preceding results will be discussed in further sections.

### Characterization, fit model and membrane-phase dependence of TCC diffusion

**Figure 3:**
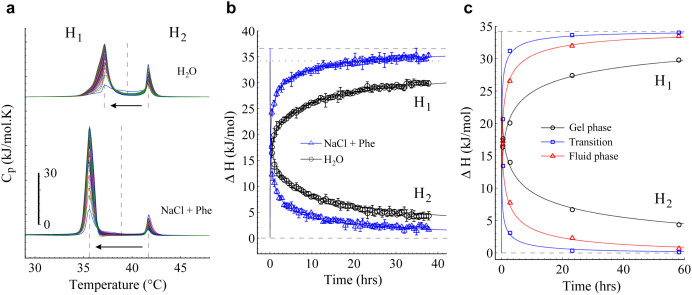
Diffusion kinetics and membrane-phase dependence diffusion of TCC. (a) A sequence of 65 calorimetric profiles of the TCC diffusion kinetics in both ‘free’ (upper curves) and ‘clinical conditions’ (lower curves). The ‘double-phase transition’ is splitted in two sections (H_1_ and H_2_) from the midpoint between the two transitions. The time between scans was about 36 min. (b) Enthalpies of H_1_ and H_2_ as function of time for both ‘free’ (black circles) and ‘clinical conditions’ (blue triangles). Best-fit models are indicated respectively, from where the diffusion coefficient, *κ*, for ‘clinical’ (2.35) and ‘free conditions’ (0.45) was obtained. Total calorimetric enthalpy values (ΔH_max_) were ~36.7 and ~34.2 kJ/mol for ‘clinical’ (dashed line) and ‘free conditions’ (dotted line), respectively. Error bars show the standard deviation. (c) Enthalpies of H_1_ and H_2_ as function of time for experiments performed in gel phase (25°C; black circles), fluid phase (41.8°C; red triangles) and phase-transition temperature (55°C; blue squares), at ‘free conditions’. The *κ* values for phase transition temperature (9.5), fluid phase (1.7) and gel phase (0.28) were obtained from the diffusion model fit. Only four representative stages from the complete kinetics were carried out to describe the membrane-phase dependence in the three respective conditions. TCC was added to MLV and twelve independent experiments were incubated the required time at their respective temperature. TCC was always used at 25 mM and the solution was adjusted to approximately pH 5 (HCl/NaOH).

To evaluate the time diffusion of local anesthetics through multiple bilayers, TCC was selected for this analysis due to its large ΔT_m_ at clinical concentrations (25 mM). A set of 65 successive heating DSC scans of TCC in MLV was obtained to analyze the diffusive kinetics. The whole diffusion kinetics in ‘clinical conditions’ is compared with a ‘free conditions’ case (free from NaCl and Phe) (Fig. 3a). From the ‘double-phase transition’, the left peak (H_1_) corresponds to TCC-perturbed membranes and the right peak (H_2_) to pure membranes not yet doped by TCC. It is clearly observed that TCC considerably induces more membrane disorder in ‘clinical’ than in ‘free conditions’. However, regardless the ΔT_m_ induced by TCC, both kinetic profiles evidence that while H_1_ increases, H_2_ decreases. After a very long time, the homogeneous distribution of TCC along the multiple bilayers leads to the disappearance of the H_2_ peak. This final state is equivalent to both the LUV-TCC case (Fig. 1c) or if TCC is added to MLV from the hydration process (Supplementary Fig. S1). Calorimetric enthalpy (ΔH), area under the curve, was calculated for each DSC scan. The total ΔH was separated in two sections from the midpoint between the two transitions, where the left peak area corresponds to H_1_ and the right peak area to H_2_ (Fig. 3b). Then, individual calorimetric enthalpies were monitored with time. The total ΔH (ΔH_max_) was always conserved: Δ*H_max_* = Δ*H*_1_ + Δ*H*_2_; where ΔH_max_ is approximately 36.7 and 34.2 kJ/mol for ‘clinical’ and ‘free conditions’, respectively. Heuristically, in the case of the H_1_ peak, a diffusion model to best fit our experimental results is: 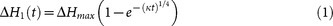
where κ is a parameter related to how fast the drug penetrates into the bilayers (for simplicity, we call it diffusion coefficient). On the other hand, from the conservation of ΔH_max_, it is easy to note that: 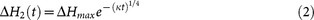
From equations (1) and (2), the best-fitted values for κ, at ‘clinical’ and ‘free conditions’, were 2.35 and 0.45, respectively (Fig. 3b). These results therefore suggest that ‘clinical conditions’ induce a faster diffusion.

To our knowledge, there have been no previous reports regarding to drug permeability/diffusion in the different membrane phases. So to inquire about the membrane-phase dependence of the TCC diffusion, we performed, in ‘free conditions’, a similar diffusion kinetic process in gel phase (25°C), phase transition point (41.8°C) and fluid phase (55°C) (Fig. 3c). Our results in MLV indicate that TCC incubation at the phase transition temperature (*κ* = 9.5) displays a faster diffusion than in fluid phase (*κ* = 1.7), which in turns is faster than in gel phase (*κ* = 0.28). These results can be explained resorting to a very well known fact: enthalpy fluctuations in membranes are maximized in the chain melting regime resulting in a pronounced heat capacity maximum. Moreover, volume and area are also known to undergo significant changes in the melting regime, mainly related to trans-gauche isomerizations of the lipid chains. From the proportionality relations between volume/area and enthalpy in the melting regime, the temperature dependence of the isothermal volume and area compressibilities have been calculated as a simple function of the heat capacity change in the transition^45^. Even both quantities have been experimentally found with pronounced maxima in the melting regime^46^,^47^,^48^,^49^. Hence, our results are well correlated with the above statement where both diffusivity and isothermal compressibilities are greatest at the phase-transition temperature (T_m_), and consecutively, higher values in fluid than in gel phase.

### The pH dependence of the TCC hydrophobicity is determinant to the ΔT_m_

**Figure 4:**
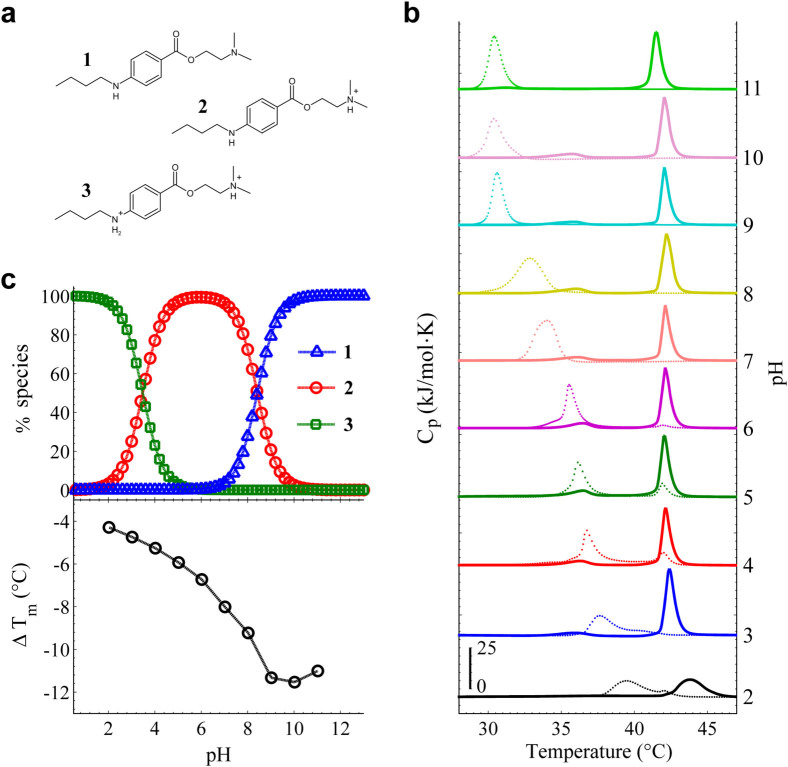
The pH dependence of the ΔT_m_. (a) Three TCC species: non-protonated (**1**), once-protonated (**2**) doubly-protonated (**3**). (b) Calorimetric profiles of both control (solid line) and TCC (dotted line) experiments as function of pH. MLV samples were prepared in different buffers depending on their pH range of buffering. The experiments were carried out in ‘free conditions’. Note that a ‘double-phase transition’ behaviour due to TCC occurs in the pH range of clinical application (pH 4–6). (c) Top panel shows the percentage of the species distribution of TCC as function of pH. When pH is above pKa_2_, specie **1** is more favored. Between pKa_1_ and pKa_2_, specie **2** is most prevailing, whilst **3** is major below pKa_1_. Lower panel shows the ΔT_m_ between the control and TCC profiles presented in b as function of pH.

Depending on pH, different molecular structures of local anesthetics can be favored^10^,^11^. TCC has two-protonation sites and therefore two pKa values (pKa_1_ = 3.4 and pKa_2_ = 8.4) (data ChemAxon); then, it exists in three different states. Figure 4a displays TCC in the non-protonated (**1**), one-protonated (**2**) and doubly-protonated (**3**) forms (data ChemAxon). In order to thoroughly evaluate how different TCC species affects the melting point depression, calorimetric assays for MLV in a wide pH range, from 2 to 11, were developed. For pH-controlling, different buffer solutions were used depending on the desired pH value. Figure 4b shows the calorimetric profiles with/without TCC as function of pH. Control experiments without TCC illustrate that buffers do not affect the DPPC main transition. Protonated species of DPPC becomes predominant below pH 1.8 (pKa) (data ChemAxon). The electrostatic repulsion of negative phosphate groups is attenuated after the lipid head group protonation, implying a stiffening of the membrane where T_m_ is increased. Our result at pH 2, T_m_ = 43.5°C, is in agreement with previous observation in DPPC^50^. On the other hand, the small decrease of T_m_ observed in control experiments at pH 11, might be attributed to an increase of the negative electrostatic potential at the phosphate groups due to the low hydrogen concentration. Such tendency of ΔT_m_ < 0 in alkaline (pH > 10) control DPPC experiments has been obtained previously^51^. Acid and alkaline hydrolysis has been reported in DPPC, however, it becomes no significant until after 1 day of incubation^52^. The ΔT_m_ decreases gradually up to pH 10–11 (Fig. 4c lower). The most hydrophobic specie of TCC (Fig. 4a, 1) is basically the most predominant above pH 10 (Fig. 4c upper). The low water solubility induces a TCC cluster formation in solution, decreasing the effective TCC concentration available for membrane interaction. Our results therefore evidence that the more the hydrophobic character of TCC, the higher the ΔT_m_. However, from Figure 4b, it is observed a small control peak (‘double-phase transition’) in the TCC experiments between pH 4–6. Different buffers were used depending on their respective range of buffering. In a first view, our results show that ‘double-phase transitions’ are only present between pH 4–6, precisely at the pH range of clinical application. Secondly, it is evident that the addition of buffer tends to significantly accelerate the diffusion process of TCC regarding to both ‘free’ and ‘clinical conditions’ (Fig. 3a, first scans).

### The intermolecular effective protonation rate (IEPR) modulates the diffusion kinetics of local anesthetics

**Figure 5:**
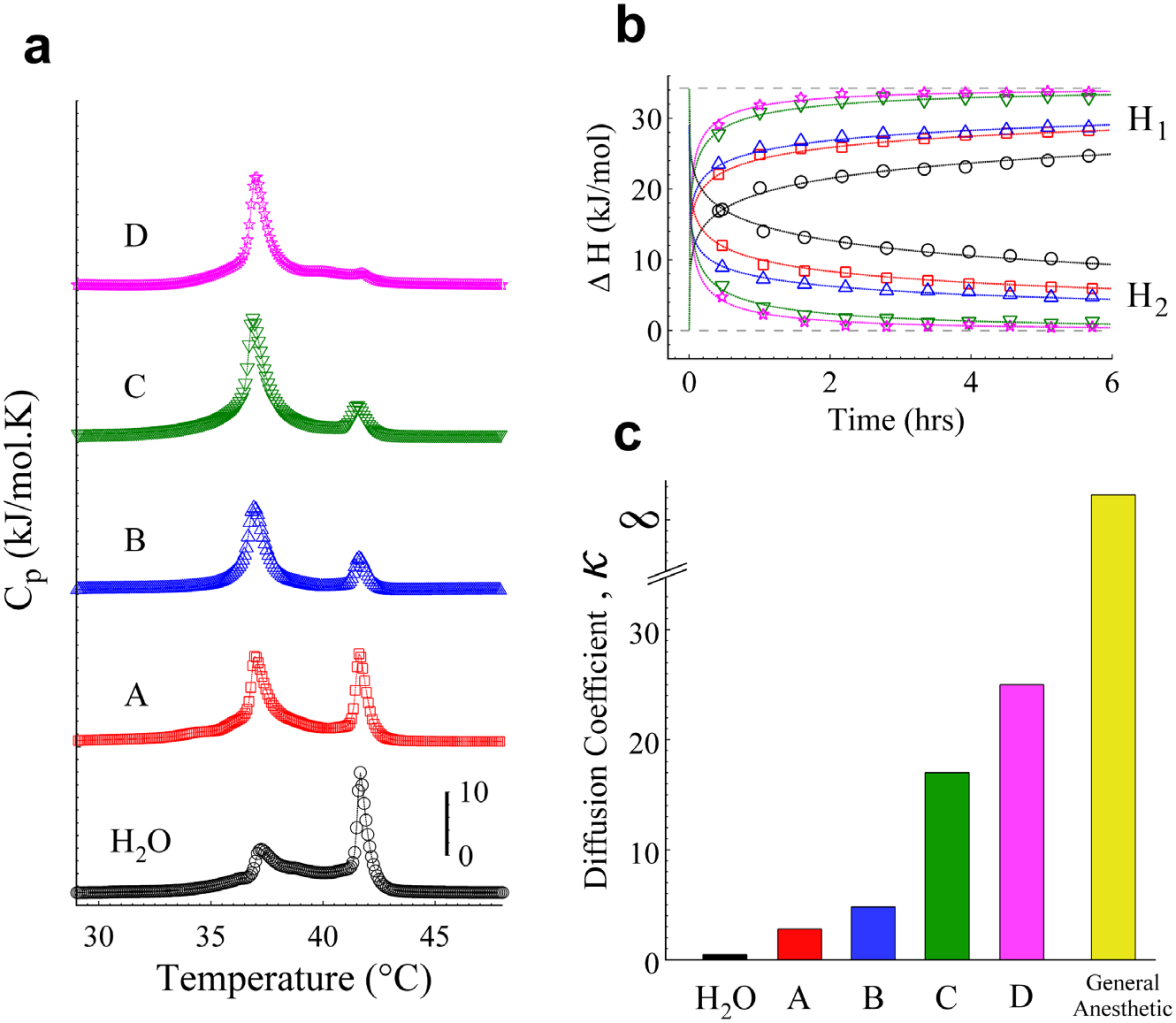
The diffusion kinetics of TCC modulated by CA. MLV liposomes were prepared in different CA solutions adjusted to pH 5 (HCl/NaOH). After 10 min of the TCC (25 mM) addition, a sequence of 10 heating scans was taken by the DSC. (a) The first scans of H_2_O (black circles), malic acid (A, red squares), citric acid (B, blue up triangles), formic acid (C, green down triangles) and glycolic acid (D, magenta stars) were sorted according to their stage in the diffusion kinetics. (b) Enthalpies of H_1_ and H_2_ as function of time for both H_2_O and the respective CA. Upper grey dashed line stands for the ΔH_max_ (~34.2 kJ/mol), which remains constant throughout the CA experiments. (c) The respective κ values were obtained from the best-fit of the diffusion model as illustrated in b. Note that the ‘single-phase transition’ obtained by general anesthetics may obey the diffusion model under a very high *κ* value.

Proton transfer plays an essential role in many biological systems^53^,^54^,^55^,^56^. Some reports have shown proton transfer rates in the order of femtoseconds - microseconds, highly depending on the chemical structure of the target molecule and its environment^57^,^58^,^59^,^60^. Recently, it has been reported slight but significant differences in the proton transfer rates in the lysosome region between normal lung cells (30 ps) and lung cancer cells (25 ps)^61^. To determine how the TCC diffusion kinetics is modulated by the IEPR, we used four weak carboxylic acids (CA) (formic, glycolic, citric and malic acid). The selected CA contains different ‘radical groups’ bonded to the carboxylic group. Since the CA are not strictly buffers, we carefully adjusted the pH to 5 before introducing the sample into the DSC equipment. This pH is a representative value of the pH range of ‘clinical conditions’, which implies a constant [H^+^] concentration. This argument therefore allows the ‘radical group’ of CA to be the free variable, since that the chemical structure of each ‘radical group’ provides to the medium a particular proton transfer rate, giving as a result an IEPR. Figure 5a displays the first scan of the respective CA experiment. It is easy to note that the ‘H_2_O’ case shows the earliest stage in the kinetic process, comprising only two-coupled equilibrium reactions (H_2_O-TCC). At pH 5, the constant interchange between species 2 (97.06%) and 3 (2.9%) of TCC is more favored than with the specie 1 (0.04%) (Fig. 4c upper). On the other hand, for the CA case, the subsequent stages are given by malic, citric, formic and glycolic acid (see Fig. 5a, A, B, C, D respectively), showing in their first scan an increasingly advanced stage of the kinetic diffusion. The CA case now corresponds to three-coupled equilibrium reactions (H_2_O-TCC-CA) with different proton transfer rates. Control experiments where carried out to illustrate that CA do not perturb the DPPC membranes (Supplementary Fig. S2).

To explore the following stages of the individual kinetics of CA in the TCC diffusion, we plotted the calorimetric enthalpies (H_1_ and H_2_) as function of time (Fig. 5b). Only 10 heating scans were enough to illustrate that the same diffusion model governs the temporal behaviors of CA. The only difference is the velocity (diffusion coefficient, *κ*) to reach the final state. To quantify the *κ* values for CA, the data were fitted using our diffusion model. Figure 5c displays the *κ* values as function of the respective CA. This result reveals that the higher *κ*, the faster the drug diffusion, and hence less time is required to achieve the final stage (‘single-phase transition’). In this way, our results with general anesthetics may conjecture a very large *κ*, since that the final stage (‘single-phase transition’) is reached almost instantaneously.

It is known that the lower the pKa value, the stronger the acid^62^. The pKa values for CA are 5.13 (malic acid), 4.67 (citric acid), 4.27 (formic acid) and 3.53 (glycolic acid) (data ChemAxon), and if water is considered as a weak acid, its pKa is about 15.7. Since acidity does not directly correlate with proton transfer rate, our results seem to outline an effect produced by the CA used in this work, where an inverse correlation between the pKa values and κ emerges: the weaker the acid, the slower the TCC diffusion.

## Discussion

The molecular arguments behind the previous results are based on the chemical nature of both local and general anesthetics. As mentioned previously, local anesthetics are weak bases with one or two protonable amine groups, which depending on pH, protonated or non-protonated species are favored^10^,^11^. General anesthetics are mainly hydrophobic^63^; inhalational are not pH dependent while intravenous have not protonable sites until extreme pH values.

Charged molecules such as protonated species of local anesthetics undergo long-range coulombic interactions. Moreover, hydrophobic molecules/atoms such as general anesthetics or hydrophobic regions of non-protonated local anesthetics are governed by short-range van der Waals interactions^64^. Supplementary Figure S3a provides a schematic representation of the coulombic and van der Waals potentials across a DPPC bilayer.

Protonated species usually exhibit a well defined position at the lipid headgroup region with a preferential orientation normal to the bilayer plane, where the charged ternary amine group maintains H_2_O-mediated coulombic interactions with the negatively phosphate groups^65^,^66^,^67^. This attractive electrostatic interaction competes against the thermal noise for lasting interactions. Potential of Mean Force calculations suggest that the interaction potential energy between the tetracaine and the membrane polar head, is approximately three times the thermal energy *K_B_T* for the charged form, whereas it is smaller than the thermal energy for the uncharged form^65^.

In contrast, the neutral form of local anesthetics appears to penetrate more deeply into the membrane, being free to diffuse in the lateral directions as well as to jump from one side of the bilayer to the other^65^,^66^. Due to the negative van der Waals potential energy in the fatty acyl core, uncharged anesthetic species are located further down in the upper part of the lipid tails with presumable perpendicular to the normal orientation. They assume however orientation parallel to the normal when they diffuse from one side of the bilayer to another^65^,^66^.

Despite hydrophobic molecules require a closer distance to interact and penetrate into the hydrophobic lipid tails, they are able to easily escape from the potential well due to thermal noise (Supplementary Fig. S3a). This suggests how the specie **1** of TCC or any general anesthetic is able to penetrate through the multiple lipid bilayers fast enough to produce a ‘single-phase transition’ profile. Nonetheless, the constant alternating switching between protonated and non-protonated species of local anesthetics impacts their lipid solubility^68^, and hence, delays enough their diffusion across the lipid bilayer to result in a ‘double-phase transition’ profile.

In the literature, proton transfer rates have been reported for water in the order of femtoseconds^69^, while for the formic acid in the order of nanoseconds^70^. To our knowledge, there are not previous evidences about the rest of the CA used in this work. Nevertheless, since proton transfer rates for water and formic acid seem to be in agreement with our results (Fig. 5a, H_2_O and C), we would expect that the other three would fit as well. Note that the medium possesses the same [H^+^] (pH 5) and the only free variable resides in the ‘radical group’ of the CA. It is well known that the strength of weak acids can be modified by the electronegativity of atoms composing the ‘radical group’. Low pKa values for strong CA may be attributed to a low electrostatic retention of H^+^ in the protonable group, explaining the acidity of the medium. This electrostatic retention of H^+^ may result in a low protonation rate. The previous supposition might also explain why the need to decrease the pH up to the pKa value increasing thus the protonated species, despite the low electrostatic retention of H^+^ of the molecule. Altogether, we conclude that the weaker the CA the higher their protonation rate.

Supplementary Figure S3b schematizes the three-coupled equilibrium reactions, which depend on ‘radical group’ of CA. The adjusted pH 5 represents the effective concentration of protons ([H^+^]) of the medium. The IEPR is expected to be function of the individual proton transfer rates from each one of the three-coupled equilibrium reactions. Thus, the mutual dependence between the IEPR and the regulation of the TCC species **2** and **3**, therefore allows the modulation of the drug diffusion. It is important to take in account that the electrostatic interaction between the positive tertiary amine of TCC, corresponding to the pKa_2_ (8.4), and the negative phosphate group of the lipid, produces a slight decrease of such pKa value^71^. Likewise, the pKa_1_ (3.4) might be shifted to either lower or higher values due to a similar electrostatic interaction. Since the CA experiments are performed at pH 5, the most relevant interchange of TCC species is carried out between **2** and **3** (pKa_1_). The pKa_1_ perturbation due to the TCC-membrane interaction is able to affect therefore the proton transfer between both TCC species (**2** and **3**). However, this effect is included in the IEPR, remaining constant in all our experiments.

Based on the supposition of that IEPR is a result of an intermolecular dependence of the participants in the medium, it would be crucial to change the concentration of any of these three participants. In Supplementary Figure S4, we present additional results varying the CA concentration (A = malic acid) in order to clarify the importance of the IEPR concept. Two different CA concentrations, below (10 μM) and above (0.1 M) the normal one (10 mM), were used to prepare the MLV liposomes. This result suggests that lower or higher malic acid concentrations considerably reduce or increase the IEPR, resulting in a faster or slower TCC diffusion, respectively. Additionally, Supplementary Figure S5 clearly shows that the TCC diffusion is also regulated by its concentration (1:1, 1:6, 1:12 mol/mol DPPC/TCC) which modifies the ΔT_m_. This analysis suggests that, effectively, varying the proportion of any of the three participants results in a different IEPR, which thus regulates the TCC diffusion.

Overall, our data demonstrate that IEPR differences play a crucial role in the diffusive kinetics of local anesthetics across multiple membranes. In comparison with general anesthetics, our results suggest that the diffusivity illustrates the inherent essence behind local or general anesthetic, which might lead to the elucidation of a more complete anesthetic mechanism and to improve the design of new drugs.

## Methods

### Reagents

DPPC was purchased from Avanti Polar Lipids. NaCl, Phe hydrochloride, PCN hydrochloride, LCN hydrochloride, TCC hydrochloride, PPF, citric acid, sodium citrate, malic acid, glycolic acid, formic acid, 2-(N-morpholino)ethanesulfonic acid (MES), tris(hydroxymethyl)aminomethane (TRIZMA), 2-amino-2-methyl-1,3-propanediol (AMPD), 2-amino-2-methyl-1-propanol (AMP) and 4-(cyclohexylamino)-1-butanesulfonic acid (CABS) were purchased from Sigma-Aldrich. Sodium salt pentobarbital was purchased from Cheminova (Pentobarbital sodium injection). All chemical substances were handled without further purification.

### MLV and LUV preparation

The desired aqueous solution comprising ultrapure water (Milli-Q-water, 18.2 MΩ.cm, pH 4–6) was used to hydrate the lipids above their melting transition (55°C). The dispersion was softly stirred at 600 rpm for 30–40 min at 55°C using a Degassing Station (TA Instruments). This procedure yields multilamellar vesicles (LMV or MLV). LUV were prepared from suspension of MLV by extrusion through 100 nm polycarbonate membranes (Nucleopore Track-Etched Membranes, Whatman), above the melting transition of lipids using a Mini-Extruder (Avanti Polar Lipids). A lipid concentration of 4 mM was used in all the experiments.

### Calorimetric analysis

Heat capacity profiles were recorded at a constant scan rate of 1°C/min and constant pressure of 3 atm. Before the samples were loaded into the DSC capillaries, the samples were degassed at low pressure (635 mmHg) for 10 min at 25°C. The calorimeter (Microcalorimeter, NanoDSC, TA Instruments) was interfaced to a PC, and data were analyzed using the software provided with the instrument. Just before starting the calorimetric scan, the samples were equilibrated for 5 min at 25°C. Most heating scans from 25 to 50°C were performed. All the DSC experiments were carried out only two times due to the high reproducibility. Details of samples preparations are described in subsequent subsections.

### Local anesthetic experiments

In both MLV and LUV preparation, 100 mM NaCl in ultrapure water was used as hydration solution. Both lipid suspensions were adjusted to standard clinical conditions by adding 73 mM PCN or 69 mM LCN or 25 mM TCC and 0.97 µM Phe^37^,^40^,^41^. Clinically, Phe is used as vasopressor in order to counteract the hypotensive effect of anesthetics^36^,^37^. The solution was always adjusted to pH ~5 (HCl/NaOH). Control experiments were carried out without anesthetics. DSC analysis was started 10 min after liposomes and the respective anesthetic came into contact (including 5 min of thermalization into the calorimeter). A sequence of ten subsequent heating scans was performed at each experiment.

### Inhalational anesthetic experiments

A self-built high-pressure system (HPS) was used to expose the liposome suspension to Xe and N_2_O. Complete details are reported in our previous work^32^. Both MLV and LUV prepared in ultrapure water were deposited into the aluminum chamber where the respective gas was introduced. The solution was always adjusted to pH ~5 (HCl/NaOH). The temperature of the samples was controlled by a water recirculating system (PolyScience) connected to the aluminum chamber. The HPS was designed to attain high pressures, allowing us to increase around 10 times the initial pressure of the chamber. Once the desired pressure was achieved, the exposure time began. The free parameters in the pressurizing process of Xe and N_2_O were the exposure time (2 hrs), temperature (70°C), and gas pressure (40 atm). Finally, once the liposome suspension was withdrawn from the HPS, the calorimetric analysis is carried out. Henry's law helps us to understand how the gases are incorporated into the aqueous suspension^32^. This law states that, at constant temperature, the solubility of a certain gas in a liquid is directly proportional to the pressure of the gas above the liquid.

### Intravenous anesthetic experiments

MLV and LUV liposomes were prepared in ultrapure water adding the respective relevant concentrations of both PPF (56 mM) and PB (25 mM). The solution was always adjusted to pH ~5 (HCl/NaOH). PPF experiments were scanned from 15 to 45°C and from 25 to 50°C for PB. Control experiments were carried out without anesthetics. DSC analysis was started 10 min after liposomes and the respective anesthetic came into contact. A sequence of 10 subsequent heating scans was performed at each experiment. However, all the calorimetric profiles were identical (‘single-phase transition’).

### Phase dependence of the diffusion kinetics

MLV liposomes were prepared in ultrapure water. The solution was adjusted to approximately pH 5 (HCl/NaOH). Different samples were incubated with 25 mM TCC at 25°C (gel phase), 41.8°C (melting temperature) and 55°C (fluid phase). Four representative stages from the complete kinetics were performed at each phase (0, 3, 24 and 60 hrs). The respective experiment was carried out under the calorimetric analysis described above.

### pH dependence of ΔT_m_

MLV liposomes were prepared in 10 mM of the following buffer solutions: glycine-HCl (pH 2), citric acid–sodium citrate (pH 3, 4 and 5), MES (pH 6), TRIZMA (pH 7), AMPD (pH 8 and 9), AMP (pH 10) and CABS (pH 11). TCC was added to samples at 25 mM, and the solutions were adjusted to the respective pH value (HCl/NaOH). Control experiments were carried out without anesthetic.

### Carboxylic acid dependence of the diffusion kinetics

MLV liposmes were prepared in 10 mM of following CA solutions: malic, citric, glycolic and formic acid. The sample was adjusted to pH 5 (HCl/NaOH) before being introduced into the DSC equipment. TCC was added to samples at 25 mM. DSC analysis was started 10 min after liposomes and the anesthetic came into contact. Control experiments were carried out without anesthetic.

## Author Contributions

R.P.I. and F.J.S.V. contributed equally: carried out all the experiments and performed the analysis of the data. Both authors contributed in the discussion of the results and preparation of the manuscript. J.C.R.S. supervised the research.

## Supplementary Material

Supplementary Information Supplementary material
