# Supplementary material for: Anesthetic Diffusion Through Lipid Membranes Depends on the Protonation Rate
Source: Sci Rep. 2014 Dec 18;4:7534. doi: 10.1038/srep07534 (PMC4269894; doi:10.1038/srep07534)
Supplement: Supplementary Information — Supplementary material [file srep07534-s1.pdf]

Anesthetic Diffusion Through Lipid Membranes Depends on the Protonation Rate

Rosendo Pérez-Isidoro, F. J. Sierra-Valdez & J.C. Ruiz-Suárez

CINVESTAV-Monterrey, PIIT, Nuevo León, 66600, México

Supplementary Information

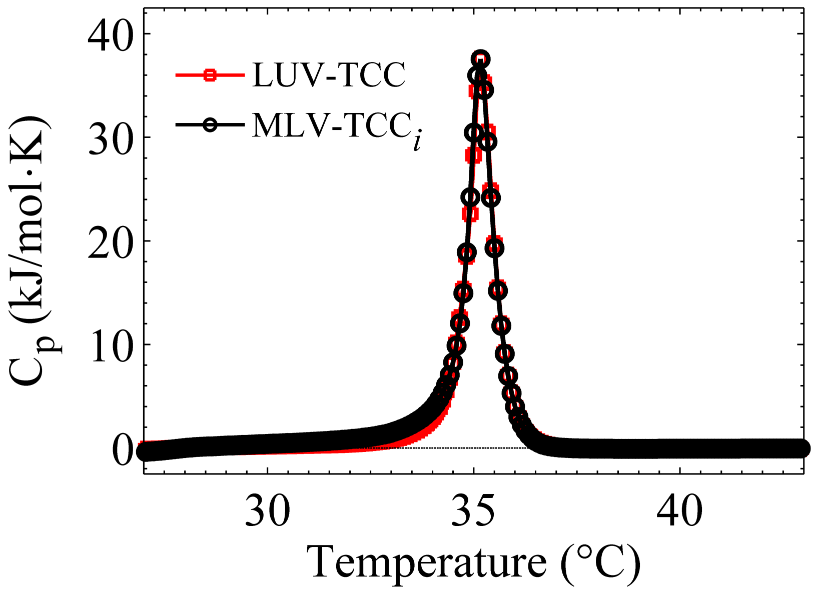

Supplementary Figure 1. Comparison between LUV and MLV$_i$ under different adding processes of TCC. In the LUV case, the addition of TCC was performed once the vesicles were formed under the standard protocol (red squares). While for the MLV$_i$, the TCC was previously mixed with the lipid solution in chloroform/methanol (2:1, v/v), and after the evaporation of the solvents, the hydration process was performed in order to form MLV containing the TCC (Black circles). In both cases, the vesicles were prepared in 'clinical conditions' in the hydration process and TCC was used at 25mM. Each experiment was carried out two times due to the high reproducibility. Error bars represent the standard deviation. It is evident that both cases present a 'single-phase transition'.

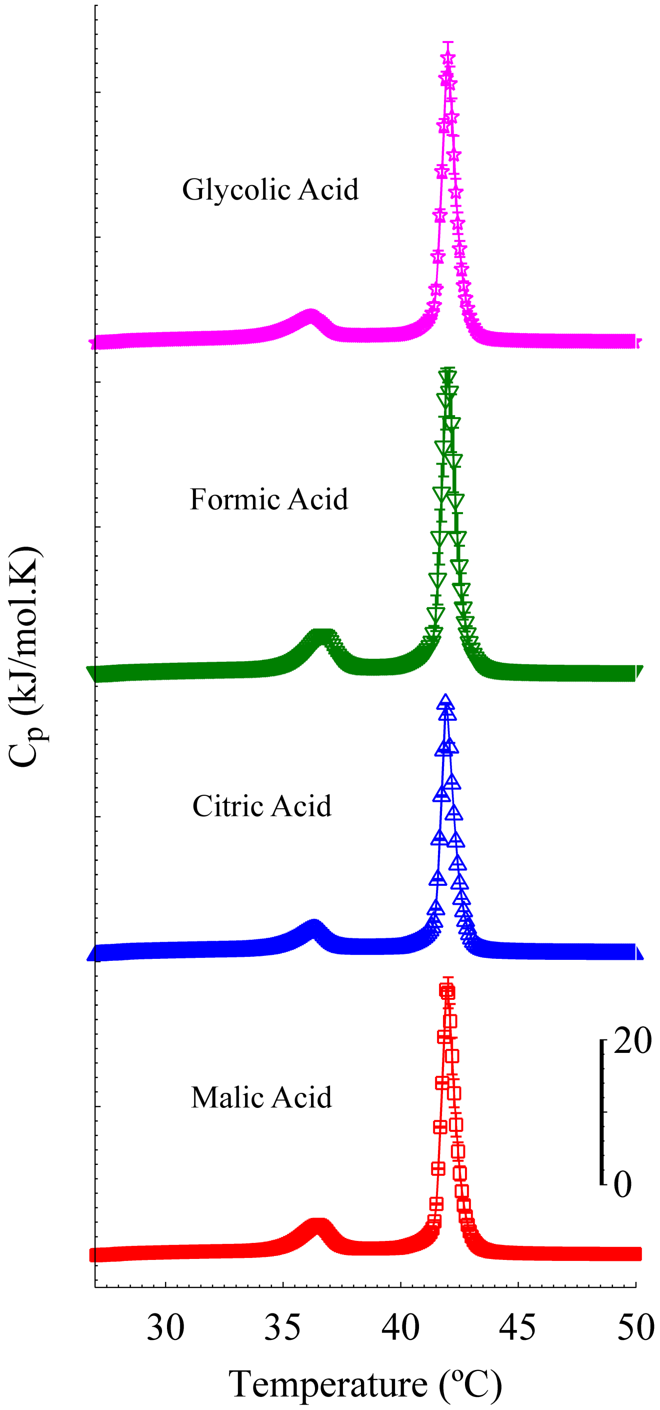

Supplementary Figure 2. Control experiments of MLV under the influence of CA. The four CA were independently tested through the hydration solution used for the MLV preparation at pH 5 without TCC. Note that CA do not perturb the MLV membranes in our conditions.

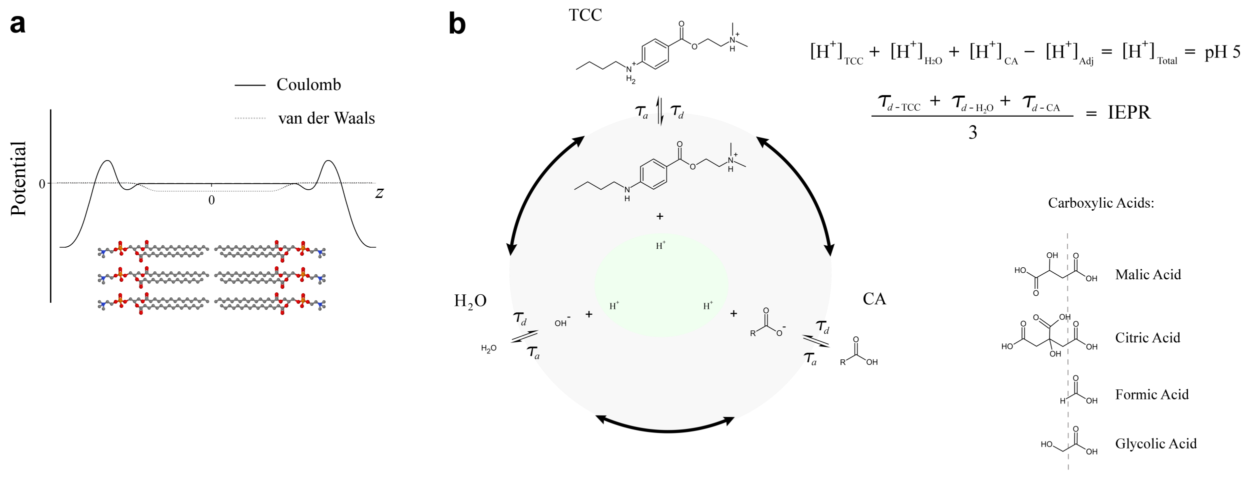

Supplementary Figure 3. Molecular speculations behind the CA influence in the protonation process. (**a**) Coulomb and van der Waals potentials across a DPPC bilayer [1]. The negatively charged phosphate group possesses a negative interaction potential while the choline group participates with a positive contribution due to their respective charges. On the other hand, the hydrophobicity of the acyl chains highlights a negative van der Waals interaction potential. (**b**) Three different coupled reactions participate in the dynamic proton transference to the medium. The contribution of $[H^+]$ in each reaction is adjusted to pH 5 using NaOH ($[H^{+}_{adj}]$), which in turn regulates the $[H^+]_{Total}$. Indeed, the IEPR may be attributed as a consequence of the mutual dependence between the enrolled reactions in which the protonation rate of the TCC is implicated. The radical group of CA is the only free variable in the medium, which may lead to a manipulation of the IEPR, which in turns regulates the TCC diffusion.

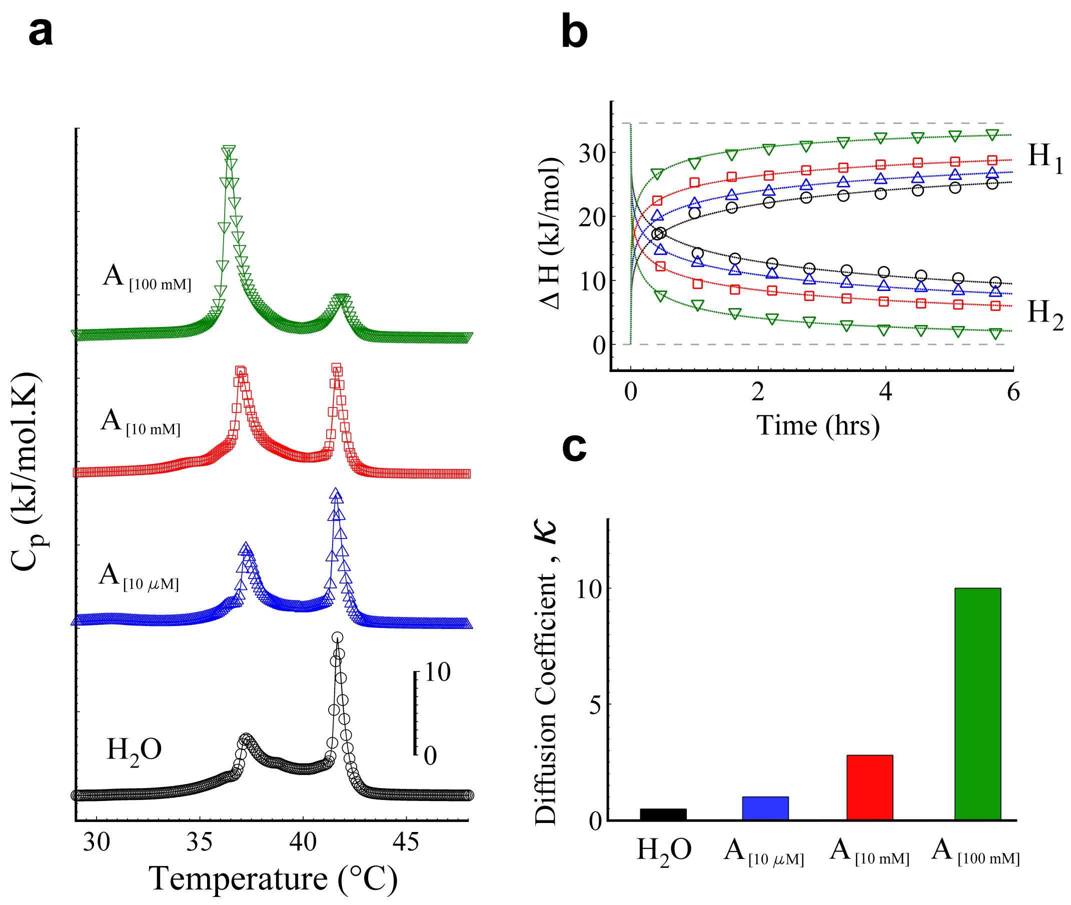

Supplementary Figure 4. TCC diffusion is regulated by the Malic Acid concentration. MLV liposomes were prepared at different Malic Acid (A) concentrations adjusted to pH 5 (HCl/NaOH). After 10 min of the TCC (25 mM) addition, a sequence of 10 heating scans was taken by the DSC. (**a**) The first scans of H_2O (black circles), Malic Acid at 10 µM (blue up triangles), 10mM (red squares), and 100mM (green down triangles) were sorted according to their stage in the diffusion kinetics. (**b**) Enthalpies of H_1 and H_2 as function of time for both H_2O and the respective Malic Acid concentration. Upper grey dashed line stands for the ΔH_{max} (~34.2 kJ/mol), which remains constant throughout the Malic Acid experiments. (**c**) The respective κ values were obtained from the best-fit of the diffusion model ass illustrated in **b**. This result suggests that lower or higher Malic Acid concentrations considerably reduce or increase the IEPR, resulting thus in a faster or slower TCC diffusion, respectively.

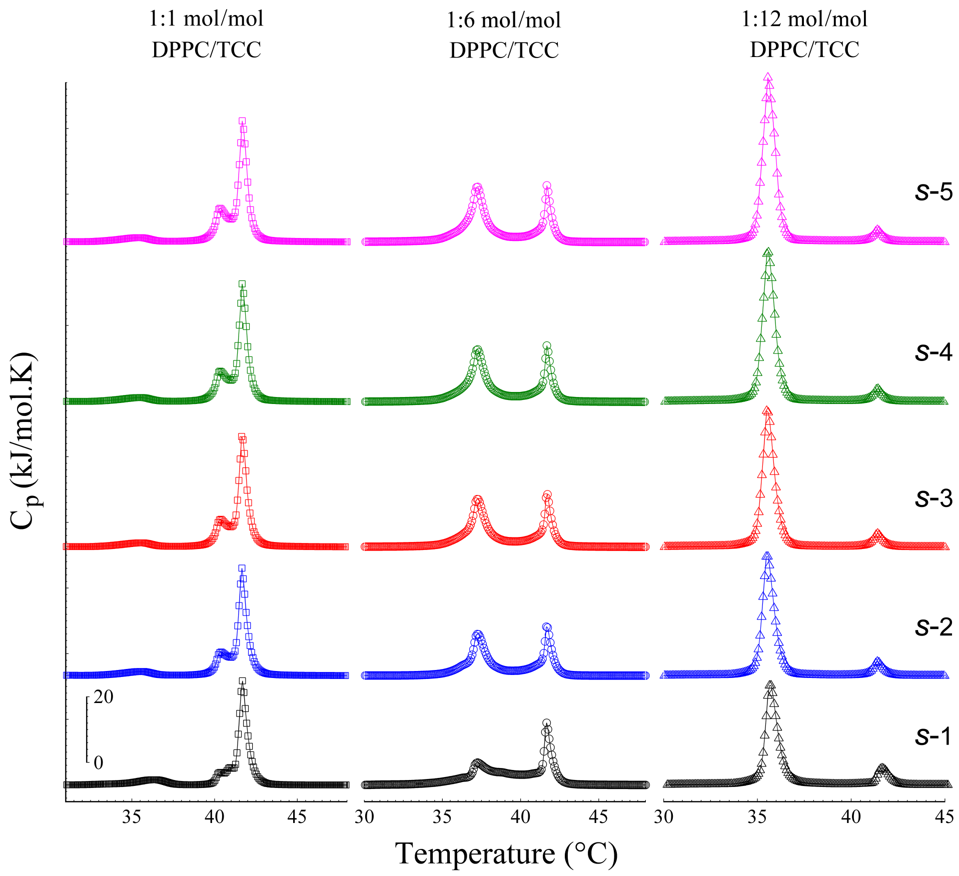

Supplementary Figure 5. TCC diffusion is regulated by its concentration. MLV liposomes were prepared in 'free conditions' adjusted to pH 5 (HCl/NaOH). After 10 min the respective TCC concentration was added, and a sequence of 5 heating scans (*s*- 1-5) were taken by the DSC. Three DPPC/TCC molar relations were study; 1:1 (~4mM TCC, squares), 1:6 (~25mM TCC, circles), and 1:12 (~50mM TCC, triangles). It is clear that the TCC diffusion is regulated by its concentration which in turns modifies the ΔT_m.

References

1. Högberg, C. J. & Lyubartse, A. P. Effect of Local Anesthetic Lidocaine on Electrostatic Properties of a Lipid Bilayer. *Biophysical Journal,* **94,** 525-531 (2008).
